# PROmotion of COvid-19 VA(X)ccination in the Emergency Department – PROCOVAXED: Study Protocol for a Cluster Randomized Controlled Trial

**DOI:** 10.21203/rs.3.rs-1405763/v1

**Published:** 2022-03-17

**Authors:** Robert Rodriguez, Kelli O’Laughlin, Stephanie Eucker, Anna Marie Chang, Kristin Rising, Graham Nichol, Alena Pauley, Hemal Kanzaria, Alexzandra Gentsch, Cindy Li, Herbie Duber, Jonathan Butler, Vidya Eswaran, Dave Glidden

**Affiliations:** UCSF Medical Center; University of Washington; Duke University; Thomas Jefferson University; Thomas Jefferson University; University of Washington; Duke University; University of California San Francisco; Thomas Jefferson University; Duke University; University of Washington; University of California San Francisco; University of California San Francisco; University of California San Francisco

**Keywords:** COVID-19, vaccine hesitancy, randomized controlled trial

## Abstract

**Background:**

We conducted in-depth interviews to characterize reasons for COVID-19 vaccine hesitancy in emergency department (ED) patients and developed messaging platforms that may address their concerns. In this trial we seek to determine whether provision of these COVID-19 vaccine messaging platforms in EDs will be associated with greater COVID-19 vaccine acceptance and uptake in unvaccinated ED patients.

**Methods:**

This is a cluster-randomized controlled trial (RCT) evaluating our COVID-19 vaccine messaging platforms in seven hospital EDs (mix of academic, community, and safety-net EDs) in four US cities. Within each study site, we randomized 30 one-week periods to the intervention and 30 one-week periods to the control. Adult patients who have not received a COVID-19 vaccine are eligible with these exclusions: 1) major trauma, intoxication, altered mental status, or critical illness; 2) incarceration; 3) psychiatric chief complaint; and 4) suspicion of acute COVID-19 illness. Participants receive an orally administered Intake survey. During intervention weeks participants then receive three COVID-19 vaccine messaging platforms (4-minute video, one-page informational flyer and a brief, scripted face-to-face message delivered by ED physicians and nurses); patients enrolled during non-intervention weeks do not receive these platforms. Approximately an hour after intake surveys, participants receive a Vaccine Acceptance survey during which the primary outcome of acceptance of the COVID-19 vaccine in the ED is ascertained. The other primary outcome of receipt of a COVID-19 vaccine within 32 days is ascertained by electronic health record review and phone follow-up. To determine whether provision of vaccine messaging platforms is associated with a 7% increase in vaccine acceptance and uptake, we will need to enroll 1290 patients.

**Discussion:**

Highlighting the difficulties of trial implementation during the COVID-19 pandemic in acute care settings, our novel trial will lay the groundwork for delivery of public health interventions to vulnerable populations whose only health care access occurs in EDs.

**Trial Status:**

We began enrollment in December 2021 and expect to continue through 2022.

**Conclusions:**

Toward addressing vaccine hesitancy in vulnerable populations who seek care in EDs, our cluster-RCT will determine whether implementation of vaccine messaging platforms is associated with greater COVID-19 vaccine acceptance and uptake in unvaccinated ED patients.

## Background

COVID-19 illness has led to over 915,000 deaths in the United States (US) as of February 15, 2022.^1^ SARS-CoV-2 vaccines are a powerful tool for mitigating the risk of acute COVID-19 illness and its’ sequelae during the COVID-19 pandemic. Efforts to mitigate the risk of COVID-19 illness by vaccination are predicated on broad acceptance of vaccines by a substantial majority of the population to achieve herd immunity.^2,3^ Vaccine hesitancy (unwillingness to receive a COVID-19 vaccine) has persisted as a major barrier to reaching this target in the US, with approximately 15% of national online survey respondents saying that they would not get a COVID-19 vaccine in the spring of 2021.^4,5^

The major limitation of most prior investigations of COVID-19 vaccine hesitancy is that they have been primarily conducted online or by telephone,^4–8^ a sampling method that may miss medically underserved and disadvantaged populations and may not reflect the attitudes of patients during true healthcare encounters.^9–11^ The emergency department (ED) setting has been commonly described by policymakers as “the safety net of the safety net”.^12^ With approximately 140 million visits in the US annually, EDs serve as the primary (and often only) health care access point for up to a fifth of the population that includes a number of vulnerable groups - immigrants, persons experiencing homelessness, the impoverished, and the uninsured, many of whom fall into high-risk categories for poor outcomes from COVID-19 infection.^13–20^ Minorities, especially African Americans and Latinos, also receive disproportionately high amounts of primary healthcare access through EDs.^16–20^ Broad, equitable COVID-19 vaccine delivery to vulnerable populations is a critical public health need that EDs are thus uniquely positioned to address.

With these principles in mind, we previously conducted the Rapid Evaluation of COVID-19 Vaccination in Emergency Departments for Underserved Patients (REVVED UP) study, consisting of surveys of medically underserved populations during ED visits at 15 geographically representative EDs across the US.^21^ We found that patients whose primary health care access occurs in EDs had greater vaccine hesitancy and particular health care access barriers, needs, and perceptions about vaccines that require specific review beyond traditional (non-ED user) community engagement techniques.

The premise underlying this research (PROmotion of COvid-19 VA(X)ccination in the Emergency Department – PROCOVAXED) is that efforts toward equitable distribution of the COVID-19 vaccine, vaccination-based herd immunity, and prevention of disease in high-risk, vulnerable populations must go where these vulnerable populations go for care – the ED. In the first specific aim toward the goal of decreasing COVID-19 vaccine hesitancy and increasing vaccine uptake in vulnerable ED populations, we conducted in-depth qualitative interviews of vaccine hesitant ED patients whose primary health care access occurs in EDs. Through these interviews, we gained actionable insight regarding reasons for COVID-19 vaccine hesitancy and other barriers to vaccination, the role of trusted messengers, and specific messaging to address hesitancy. We then used these findings to develop population-specific COVID-19 vaccine (PROCOVAXED) messaging platforms (videos, informational flyers, and scripts for face-to-face ED provider messaging) that addressed their specific COVID-19 vaccine concerns.

The objectives of this trial are to determine 1) whether implementation of PROCOVAXED trusted messaging platforms in EDs is associated with increased COVID-19 vaccine acceptance (the converse of vaccine hesitancy) in ED patients at the time of their ED visit; and 2) whether implementation of PROCOVAXED platforms in EDs is associated with increased COVID-19 vaccine uptake in unvaccinated ED patients (30 to 32 days after their index ED visit). Our central study hypothesis is that Implementation of PROCOVAXED messaging platforms in EDs will be associated with greater COVID-19 vaccine acceptance and uptake in unvaccinated ED patients. Herein, we present our trial’s rationale, methodology, and study procedures.

## Design

This is a cluster-randomized controlled trial (ROT) of implementation of our multimedia COVID-19 messaging platforms in seven hospital EDs (mix of academic, community, and safety net EDs) in four US cities: 1) San Francisco, CA: Zuckerberg San Francisco General Hospital [ZSFGH] and UCSF Medical Center – Parnassus; 2) Philadelphia: Thomas Jefferson University Hospital, Methodist Hospital and Jefferson Torresdale Hospital; 3) Seattle, WA: Harborview Medical Center; and 4) Durham, NC: Duke University Medical Center.

## Rationale for Cluster Design

Our primary goal with this research is to determine whether implementation of PROCOVAXED as an ED-site level intervention results in greater acceptance and uptake of COVID-19 vaccines in vulnerable ED populations. Each site sees approximately 125–250 patients per day and applying or not applying the intervention (delivery of PROCOVAXED messaging) under an individual patient randomization scheme in this high workflow, rapid patient turnover ED environment is less practical and would likely result in extensive cross-contamination between intervention and control arms. Therefore, randomization by weeks at sites and removal of the intervention from the site completely during specified time periods of non-intervention was considered to be the optimal approach. Although a single switch of the intervention at each site (i.e., stepped-wedge trial design) is easier to enact, changes in general population attitudes overtime limiting the validity of this trial method. We expect changes in baseline acceptance of the COVID-19 vaccine over time, which would likely introduce substantial bias toward or against the intervention. These practical and methodological benefits of the week unit cluster RCT far outweigh the smaller sample size and easier analysis with an individual patient unit RCT or a stepped-wedge design.

## Randomization Plan

Randomizations are computer-based pseudo-random sequences of seven-day (one week) periods. Within each of the seven study sites, we randomized 30 one-week periods to the intervention group and 30 one-week periods to the control group to ensure equal allocation to control and intervention settings. We stratified sequences by study week period so that three centers will be in the control condition for one week and four centers will be in the experimental condition for one week, or vice versa in a Latin square design. This is intended to minimize the effect of secular trends on the comparison of the intervention. We generated a 60-week study calendar based on this randomization scheme. To maintain masking of allocation, sites are notified of their treatment assignment for the next week no more than three days prior to that week.

## Study Enrollment Procedures

Practical budget considerations and limits on research personnel in patient care areas during the COVID-19 pandemic preclude 24/7 study enrollment and delivery of the study intervention. Thus, we will enroll a convenience sample of patients across all study sites, approaching all potentially eligible adult patients who present to study EDs during 6 to 10-hour weekday blocks, typically beginning at approximately 09:00 and continuing to approximately 17:00. Sites have leeway to choose their preferred daytime enrollment block periods, as long as those blocks remain consistent between study arms throughout the study. Research staff avoid telling providers whether this is an intervention versus control period.

All sites have ED dashboards that include patient age, chief complaint and COVID vaccination status. Research staff review these dashboards, query ED providers regarding suitability for the study and approach patients who potentially meet inclusion/exclusion criteria. We include adult (> 17 years of age) patients presenting to study sites according to the following inclusion criteria: 1) not already vaccinated for SARSCOV2; 2) able to provide informed consent; 3) fluent in English or Spanish (inclusion of Spanish speakers will only occur at three sites that have Spanish-speaking research staff); and 4) anticipated ability to complete study intervention in ED, i.e., able to watch the short video. We exclude patients with the following characteristics: 1) inability to participate in a survey because of major trauma, intoxication, altered mental status, or critical illness; 2) in police custody or incarceration; 3) psychiatric chief complaint or on psychiatric hold; 4) medical reason (as per the ED provider or patient) that they cannot receive a COVID-19 vaccine (e.g., instructed by their primary physician that they should not receive a COVID-19 vaccine); and 5) suspicion of acute COVID-19 illness with any of the following constituting suspicion: cough, fever, myalgias, shortness of breath, sore throat, chest pain and patient or provider declaration of suspicion of acute COVID-19. Of note, given that many patients are receiving COVID-19 testing for surveillance reasons (e.g., routine admission testing), performance of a COVID-19 test itself is not an automatic exclusion. However, if a COVID test returns a positive result, the patient is excluded.

For potential study patients, we deliver scripted verbal consent for two short study surveys (the Intake Survey [see Additional File 1] and the Vaccine Acceptance Survey [see Additional File 2 & 3]) in a manner that we have used with numerous other ED survey studies, including those that have addressed COVID-19 vaccine hesitancy.^21^ Considering that the intervention (COVID-19 vaccine messaging) is entirely educational and firmly a part of standard best-practice ED care (COVID-19 messaging is currently enacted in EDs across the US), only verbal consent is required. Patients are informed that they will not be compensated for participation.

## Survey Administration

For patients agreeing to the above surveys and meeting inclusion/exclusion criteria, the Intake Survey is administered to assess demographics and other study subject characteristics. All surveys are delivered orally - research staff read questions to the participants in their preferred language and record responses.

Approximately one hour after the Intake Survey, the Vaccine Acceptance Survey is administered. Although we are using an hour as our general guide for this Vaccine Acceptance Survey, we expect variability in patients’ visit time and care plans in the ED (e.g., patients may be undergoing procedures or away from their rooms for x-rays precluding the survey at one hour). Therefore, research staff can conduct this survey anytime between 30 minutes and six hours after the Intake survey. The Vaccine Acceptance Survey for Intervention [see Additional File 2] arm participants assesses whether the messaging platforms affected their views on getting a COVID-19 vaccine. The Vaccine Acceptance Survey for Non-Intervention [see Additional File 3] arm participants asks whether anyone delivered messages about COVID-19 vaccines to them in the ED. The last question in the Vaccine Acceptance Survey in both arms of the study is *“ Would you accept the COVID vaccine in the emergency department today if your doctor asked you?”*

For all subjects saying ***“**No”* to the question *“ Would you accept the Covid vaccine in the emergency department today if your doctor asked you?”*, research staff ask if they can contact them by phone and review their electronic health records (EHR) in a month for follow up, with options to agree to both phone calls and EHR review, only phone call (no EHR review), only EHR review (no phone call). If the participant agrees to follow-up, then the CRC will obtain the relevant full written informed consent, including separate FIIPAA document agreements. They then ask subjects for their best phone number(s) to reach them for a follow-up phone call. They also ask for 1-month follow up in those subjects who said *“Yes”* to accepting the COVID-19 vaccine in the ED but did not it in the ED.

## Study Intervention

The intervention consists of three COVID-19 messaging platforms that were developed by our team in the first phase of this work, using findings from qualitative interviews focused on understanding vaccine hesitancy and on potential methods to addressing this hesitancy.

1. Videos: Short (approximately 4-minute) Public Service Announcement type videos that are presented on an electronic tablet. We have developed five versions; all with the same wording in the message, but each with a different pair of physician messengers:
  - African American physicians
  - Latinx physicians, English version
  - Latinx physicians, Spanish version
  - Mixed race physicians
  - White physicians
2. Printed materials: One-page information flyer. We have developed five versions; all with the same format and wording/captions in the flyer, but each with different pictures of patients receiving the vaccine and health care providers administering the vaccines.
  - Predominantly African American patients and providers
  - Predominantly Latinx, English version patients and providers
  - Predominantly Latinx, Spanish version patients and providers
  - Mixed race patients and providers
  - Predominantly White patients and providers
3. Face-to-face messaging: A short (<1 minute) scripted message printed on a sheet of paper and delivered by one of the patient’s providers in the ED (physician, nurse or mid-level practitioner).

At the end of the Intake Survey, research staff ask patients if they are willing to watch a short video about COVID-19 vaccines. If they agree to watch the video, research staff show them a video on the electronic tablet. After finishing with the video (or after refusal to watch the video), the research staff ask the patient if they would like to see an informational flyer about COVID-19 vaccines. If the patient agrees, then staff hand the patient the flyer. Staff then ask the subject if they may return in approximately an hour for the Vaccine Acceptance Survey. After leaving the participant’s room, staff ask one of the patient’s primary providers (doctor, mid-level practitioner, or nurse) to deliver the COVID-19 face-to-face vaccine message, using the scripted message.

We deliver messaging from our platform libraries in patients’ preferred language (English or Spanish only). In our previous qualitative interviews, patients reported preferences for ideal vaccine messengers as being congruent with their race and ethnicity. Thus, research staff match videos and informational flyers with subjects’ declared ethnic and racial characteristics declared during the Intake Survey (e.g., Latinx messenger on video with Latinx subject). All surveys and interventions are delivered in real-time patient visits in site EDs, during waiting times such that they do not interfere with patient care.

## Description of Usual Care

Study procedures during Control Period (Non-Intervention) blocks are identical to procedures in Intervention blocks with the exception that patients are not given the Intervention. Randomization to the control group does not in any way preclude delivery of vaccine messaging by ED providers, and research staff are not telling providers to avoid delivering vaccine messaging. During control group weeks, ED providers are free to practice their usual practice of delivering or not delivering vaccine messaging.

**Figure 2:**
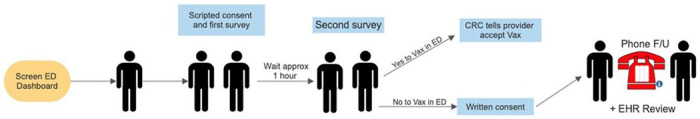
Control Period Blocks Study Flow

The anticipated flow of the study during **Control Period Blocks** is summarized in Fig. 2.

## Provider Notification of COVID-19 Vaccine Acceptance

At this time, all of our EDs have the capability of administering COVID-19 vaccines in some manner, and we expect that this vaccine availability will continue for at least the first six months of the trial. The last question in the Vaccine Acceptance Survey in both arms of the study is *“Would you accept the COVID vaccine in the emergency department today if your doctor asked you?’* When a participant says they will accept the vaccine, research staff ask the patient if they can notify the patient’s providers that they said they will accept the vaccine. They also ask the participant if research staff can check to see if they receive the vaccine in the ED. Other than this question and notification, research staff do not push that they get vaccinated. They do not provide any counseling and do not tell patients whether they qualify for a COVID-19 vaccine in the ED.

When patients agree to the vaccine and agree that research staff can notify the ED providers of vaccine acceptance, research staff notify the provider of vaccine acceptance. We are clarifying with providers that we have not reviewed their medical history, indications and contraindications to COVID-19 vaccination. Research staff emphasize with both patient and provider that it is up to the provider to determine whether the patient can receive the vaccine in the ED (research staff are merely informing providers that the patient would accept it if offered). Staff later check with the provider and patient to see if the patient received the COVID-19 vaccine in the ED.

## Data entry and management

We manage data using REDCap, hosted by the core site (UCSF), for secure data entry and management. Research staff have the option of inputting survey responses to the REDCap database on iPads in real time or using paper surveys (and later inputting into REDCap). For study subjects who have consented to phone and EHR follow-up, separate files linking patient identifiers (medical record numbers and phone numbers) to unique study ID numbers are housed at individual study sites in files, separate from other study data. We developed a detailed data dictionary to ensure consistent standards across sites and to reduce missing or erroneous data using the REDcap data quality tool.

## Primary Outcomes and Ascertainment

Our primary outcome of acceptance of a COVID-19 vaccine in the ED is ascertained in both arms of the study by the question in the Vaccine Acceptance Survey,” *Would you accept the COVID vaccine in the emergency department today if your doctor asked you?’*: “Yes” to this question = acceptance; “No” or “Unsure” = non-acceptance.

Our primary outcome of COVID-19 vaccine uptake 32 days after their index ED visit is ascertained via 1) confirmation of receipt of a COVID-19 vaccine during their index ED visit, 2) review of EHR for receipt of a COVID-19 vaccine at 28 days, and 3) phone follow-up at 28 to 32 days - response to the question *“Have you received a COVID-19 vaccine since you were in the Emergency Department?*

## Statistical approach

This is a superiority trial in which we seek to verify our central study hypothesis that provision of PROCOVAXED will result in greater acceptance and uptake of the COVID-19 vaccine. Following the recommendations of Hussey and Hughes^22,23^, our statistical analyses will focus on comparing the vaccine uptake rates during intervention periods and control periods using mixed effects logistic models. The outcomes of interest are the binary indicators of whether a patient will accept the COVID-19 vaccine (*“Will you accept the COVID-19 vaccine if it was offered to you”* – yes/no) and whether they have received a COVID-19 vaccine (uptake - yes/no) upon follow-up at 30 to 32 days. Models will include a random center effect to accommodate potential within-center characteristics (e.g., case mix, demographics), as well as terms for time and intervention. Hypothesis tests will focus on the statistical significance of the intervention indicator. We will fit the mixed effects models using maximum likelihood and routines in Stata.

We will test our primary hypothesis and analyze outcomes according to the study arm (index visit in intervention time period vs control time period) to which patients were allocated, regardless of whether they received PROCOVAXED messaging platforms or not – an *intention to treat analysis*. We will also conduct a *per-protocol analysis*, in which we assess results that would occur if they actually did or did not receive PROCOVAXED messaging (e.g., viewed the video clip given to them) during their index visit (ascertained by direct questioning in the Vaccine Acceptance Survey). When compared to the primary analysis, the per-protocol analysis will allow us to dissect the reasons for success (or failure) in demonstrating improved vaccine acceptance and uptake with PROCOVAXED. For example, if we find better acceptance and uptake in the per protocol analysis and not in the intention to treat allocation analysis, we would subsequently seek ways to improve delivery of PROCOVAXED messaging. Conversely, if both analyses fail to improve acceptance, then the PROCOVAXED intervention truly fails and other efforts to improve delivery would not be indicated.

In addition to the effects on *total* vaccine acceptance, we will also examine the effect of PROCOVAXED on acceptance in patient sub-groups, especially African Americans, Latinos and patients who lack primary care (as determined by direct questioning). We will additionally stratify outcomes by study site (representing different regions of the country and different communities), age, gender, race/ethnicity, as well as patient-level experience characteristics, such as having had COVID-19.

We will also analyze data from Intervention group Vaccine Acceptance Survey assessing participants’ views on the vaccine messaging platforms. This data includes opinions on which platforms were helpful in promoting vaccine acceptance and feedback on improving the platforms.

## Sample Size Considerations

The sample size calculations for this research are governed by testing the hypothesis that implementation of a trusted messenger informational program will be associated with increased acceptance and uptake of COVID-19 vaccines in unvaccinated ED patients. Considering the commonality of hesitancy (non-acceptance), the high benefit of increasing acceptance and the negligible risk of the intervention (a trusted messaging program), even a small effect size of increased acceptance would be a clinically important difference. By investigator consensus and in consultation with a panel of health policy experts, we have determined that vaccine messaging platforms would be clinically useful if they increased acceptance by 7%. Similarly, with the same considerations of negligible risk, we determined that these platforms would be useful with an effect size on vaccine uptake of 7%.

Our sample size calculations accommodate the randomization of clusters design consisting of one-week periods (PROCOVAXED platform weeks versus non-intervention weeks) to the intervention at each of seven sites. To avoid period effects, we will assign sites using a Latin square design S2. We base the sample size calculation on the comparison of the proportion of patients who accept the vaccine between the PROCOVAXED and usual care time periods using standard formulae for individual randomization. We have verified that these sample sizes are conservative by simulation of data using a mixed random effects model.

When our protocol was originally written in February 2021, vaccines were not widely available and the degree of baseline vaccine acceptance was unknown. We therefore calculated sample sizes for a wide range of vaccine acceptance and uptake rates with a plan to measure these in the non-intervention (control) group during the first month of the trial. After the first month of the study, we estimated that our baseline vaccine acceptance and uptake rates (without intervention) will be approximately 15%. With this baseline uptake rate of 15%, we find that at an alpha = 0.05 level and a power of at least 0.9, we will need to enroll 1,290 patients (645 in each arm) in the study to detect the difference of interest (a setting in which the vaccine acceptance rate will increase by 7% in PROCOVAXED weeks). With this same baseline 15% rate of uptake and the same specifications for power, we will need to enroll 1,290 patients (645 in each arm) to detect a vaccine uptake difference of 7%. Thus, our target enrollment for this implementation trial is 1290 subjects across all sites.

In terms of total projected time for enrollment, we expect enrollment of four subjects per site per week at the seven sites, or 28 enrollees per week. We therefore expect to attain our target enrollment of 1290 subjects in approximately 46 weeks.

## Early Termination of Study Monitoring Committee (ETSMC)

In our study we seek to determine whether implementation of COVID-19 vaccine messaging platforms in the ED result in greater COVID-19 vaccine acceptance and uptake in unvaccinated ED patients. One of the primary goals of the data safety and monitoring boards for most studies is to prevent the ongoing use of unsafe treatments. This safety goal does not apply to this study for the following reasons: 1) We are not delivering a drug or other physical intervention in this trial; the intervention is vaccine messaging. 2) We are not testing or measuring the safety of any drug or therapy – COVID-19 vaccines have undergone rigorous testing in multiple other studies; 3) The intervention (vaccine messaging) is an accepted and highly recommended public health intervention in all patient care settings; 4) Randomization to non-intervention week does not preclude delivery of vaccine messaging by providers. Providers are unaware of treatment arms during the study, and we are not telling providers to avoid delivering vaccine messaging. During non-intervention weeks, providers are free to practice their usual practice of delivering or not delivering vaccine messaging; 5) We are not telling patients that they qualify for the COVID-19 vaccine in the ED. We are only asking this question:” *Would you accept the COVID vaccine in the emergency department today if your doctor asked you?”*; and 6) We are not prescribing or ordering vaccines in study patients. We are merely informing ED providers that their patient would accept the vaccine if they were offered it in the ED. We are emphasizing with providers that we have not reviewed their medical history, indications and contraindications to COVID vaccination. The decision as to whether they would offer or give the vaccine is entirely left up to the ED provider.

Given the above-described rationale about safety, there remain two primary considerations with regard to stopping the trial before sample size enrollment in this study:

1. Decreased vaccine acceptance or decreased vaccine uptake in the intervention arm – It is possible that the intervention may increase vaccine hesitancy (decrease vaccine acceptance and uptake) and that this ineffectiveness could be determined statistically before full patient enrollment. Under this circumstance, continuation of the trial would therefore be futile and not ethically justified.
2. Superior efficacy of the intervention – Conversely, it is also possible that the intervention may clearly improve vaccine acceptance and uptake before full sample size enrollment. In this case, continuation of the study in the non-intervention arm would no longer be justified.

*T*o assess for either of the two early termination scenarios, we have established a three-person ETSMC to conduct a blinded interim analysis at the one-quarter, one half, and three-quarter points of study enrollment (after enrollment of 323, 645, and 977 patients). We will provide the ETSMC a detailed algorithm with clearly identified criteria for this early termination assessment.

## Site Orientation and Manual of Operating Procedures

We developed orientation materials to familiarize the ED Sites with the study protocol. Each site employs one or more RCs, who report to the site principal investigator (PI) and are responsible for day-to-day study implementation. We developed and disseminated a manual of operating procedures (MOP) with standard personnel training methods, including education kits with scripts, summary cards, and PowerPoint presentations to assist coordinators in the orientation of site clinicians and other staff to our study protocol. We convened videoconference calls to review this summary and develop plans for optimization of study procedures to improve usability and workflow. We continue to update the MOP to reflect changes in study procedures.

We reviewed study implementation procedures with sites individually and at group conferences prior to study intitiation. We conducted walk-through sessions of workflow on hypothetical study subjects in both Intervention and Non-intervention study arms. We continue to refine procedures with updates delivered to site PIs and research staff during weekly videoconferences. We maintain a study hotline during primary study hours and encourage study personnel to contact the PI and Central Study Coordinator for all issues and queries.

We implement rigorous methods for clinical trial quality assurance and performance improvement, including: 1) systematic review of enrollment logs, 2) quarterly audits of random samples of data for accuracy and missing elements, and 3) structured review of protocol deviations or violations. The Central Study Coordinator prepares monthly summary report cards, tabulating individual site quality assurance metrics for review during scheduled Steering Committee calls. The study PI discusses site-specific data with site PIs individually and summarizes these data collectively during Steering Committee calls, with prompt dissemination of plans for process improvement.

## Discussion

Emergency departments provide both acute care and vital public health services to large swaths of the US population, especially disadvantaged populations who lack primary care. In our previous research, we identified a critical need to address COVID-19 vaccine hesitancy and uptake in vulnerable populations who primarily seek care in the ED. In this trial we seek to address the critical need for COVID-19 vaccine messaging and access, testing the hypothesis that implementation of COVID-19 messaging platforms in EDs will improve vaccine acceptance and uptake in unvaccinated ED patients.

## Expected Key Results

In addition to determining whether implementation of COVID-19 messaging platforms in EDs improves vaccine acceptance and uptake in the general population of unvaccinated ED patients, we will assess the efficacy of messaging platforms in a number of other subpopulations. Given that PROCOVAXED messaging may work for some patient sub-groups and not others, these additional analyses will guide targeted messaging. Data on participants’ views regarding the three different messaging platforms will guide future modifications of vaccine messaging.

## Strengths and Limitations

Our research is particularly innovative in a number of ways, and may set a new paradigm for public health interventions to vulnerable populations, including messaging for other vaccinations like influenza, through the ED. The sheer number of ED visits across the country afford our research very high impact. If our intervention increases vaccine acceptance and uptake in 7% of vaccine hesitant patients, this could potentially lead to the delivery of tens of thousands of COVID-19 vaccines to people who would not otherwise get vaccinated.

Perhaps our greatest limitation in this research is the limited pool of unvaccinated patients overtime in our EDs. When we began this work in December 2020 to March 2021, vaccine hesitancy was expressed by over 40% of the populations in our EDs. As of January 2022, national and local efforts have led to very high rates of vaccine uptake in our cities, ranging from 85 to 94%. While emphasizing that high vaccination rate is a great thing, we now have a limited pool of unvaccinated patients in our EDs, who may be particularly steadfast in their views and resistance to COVID-19 vaccine messaging.

Our work highlights the difficulties of performing an RTC during a pandemic in the acute care setting where most acutely ill COVID-19 patients receive care – the ED. Surges in the pandemic, particularly with the Omicron variant, may make enrollment difficult. At our study sites as many as a third of all patients in the ED during January 2022 were either under suspicion for COVID-19 or tested positive for COVID-19 in the absence of symptoms; these patients therefore were excluded from consideration for the study. Additionally, the surge has led to substantial ED boarding of admitted patients, in turn leading to major decreases in patient turnover in the ED. These factors may lead to much slower enrollment and longer time to reach our sample size than originally anticipated.

Research staffing to conduct the in-person study procedures in the ED also presents significant challenges. While all research staff have received COVID-19 vaccines and boosters, research team safety and avoiding undue exposure are top priorities. With high levels of acute COVID-19 infections and need for quarantine protocols among clinical and research staff at our institutions during surges, we expect that there will be days in which we will not be able to conduct the study.

## Trial Status

All study procedures were approved by the UCSF Committee on Human Research as a central institutional review board (protocol #21-34004; initial approval 4/27/21, final revision approved 12/2/21), with multisite reliance mechanisms in place at the non-UCSF sites. We have registered our trial in the Clinicaltrials.gov depository (https://clinicaltrials.gov/ct2/show/NCT05142332). We began enrollment on December 6, 2021. Unless early termination criteria are met, we expect to continue enrollment through December 2022.

## Conclusions

We have identified a critical need for messaging to address COVID-19 vaccine hesitancy in ED patients. At seven safety net emergency departments, the PROCOVAXED cluster-RCT will test the hypothesis that Implementation of PROCOVAXED messaging platforms in EDs will be associated with greater COVID-19 vaccine acceptance and uptake in unvaccinated ED patients.

## Supplementary Material

Supplement 1

Supplement 2

Supplement 3

## Figures and Tables

**Figure 1:**
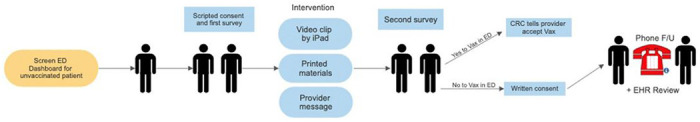
Intervention Blocks Study Flow

## Data Availability

available upon request
