# Supplementary material for: PROmotion of COvid-19 VA(X)ccination in the Emergency Department – PROCOVAXED: Study Protocol for a Cluster Randomized Controlled Trial
Source: Res Sq. 2022 Mar 17:rs.3.rs-1405763. Preprint. [Version 1] doi: 10.21203/rs.3.rs-1405763/v1 (PMC8936116; doi:10.21203/rs.3.rs-1405763/v1)
Supplement: Supplement 1 [file 5eeb82adcb969b0181da3952.docx]

**PROCOVAXED FIRST SURVEY (SAME FOR INTERVENTION AND NON-INTERVENTION BLOCKS)**

**Subject ID _________________ Date and Time: ___________ CRC Initials:_________**

|  | | |
| --- | --- | --- |
| **#** | **Questions** | **Answer(s)** |
| **1.** | Have you previously been diagnosed with COVID-19 or had a positive COVID-19 test? | 1. Yes 2. No 3. Unsure |
| **2.** | Have you had a Covid vaccine? | 1. Yes 2. No 3. Unsure |
| **2A** | If Yes, where did you get it? | 1. My doctor’s office or clinic 2. A pharmacy 3. An ER - emergency department 4. An urgent care clinic (not my doctor or clinic) 5. Other________________ 6. I don’t remember |
| **3.** | Have you had a flu vaccine in the past 4 months? | 1. Yes 2. No 3. Unsure |
| **3A** | If Yes, where did you get it? | 1. My doctor’s office or clinic 2. A pharmacy 3. An ER - emergency department 4. An urgent care clinic (not my doctor or clinic) 5. Other________________ 6. I don’t remember |
| 4. | How old are you? | 1. __ __ __ |
| 5. | What is your gender? | 1. Male 2. Female 3. Transgender male 4. Transgender female 5. Non-binary/gender non-conforming 6. Not listed ________________ 7. Prefer not to answer |
| 6. | Are you of Hispanic or Latino Origin? | No  Yes  Prefer not to answer |
| 7. | Which best describes your race? Check all that apply. [OMB/HHS Standards] | - 1. American Indian/Alaska Native   2. Asian [🡪SEE BRANCHING LOGIC]   3. Black or African American   4. Native Hawaiian/Other Pacific Islander [🡪SEE BRANCHING LOGIC]   5. White   6. Some other race (specify): ____________________ |
|  |  | If Asian, please specify which sub-category (select as many as apply)   1. Chinese 2. Filipino 3. Asian Indian 4. Vietnamese 5. Korean 6. Japanese 7. Other Asian (e.g., Pakistani, Cambodian, Hmong) |
|  |  | If Native Hawaiian/Other Pacific Islander, please specify sub-category (select as many as apply)   1. Native Hawaiian 2. Samoan 3. Chamorro 4. Other Pacific Islander (e.g., Tongan, Fijian, Mashallese) |

| Housing / Vivienda (AHC HRSN Screening Tool; <https://innovation.cms.gov/files/worksheets/ahcm-screeningtool.pdf>) (source: CMS/AHC) | | | |
| --- | --- | --- | --- |
| **#** | **Questions** | | **Answer(s)** |
| 8. | What is your living situation today? | 1. I have a steady place to live (home, apartment or other) 2. I have a place to live today, but I am worried about losing it in the future 3. I do not have a steady place to live (I am temporarily staying with others, in a hotel, in a shelter, living outside on the street, on a beach, in a car, abandoned building, bus or train station, or in a park | |

| *Access to Care / Acceso a Cuidados (NHANES/BRFSS/AHC CMS)* | | | |
| --- | --- | --- | --- |
| **#** | **Questions** | | **Answer(s)** |
| 9. | Do you have any kind of health care coverage, including health insurance, prepaid plans such as HMOs, or government plans such as Medicaid, Medicare, or Indian Health Service? | CHECK ALL THAT APPLY   1. I do not have health insurance 2. Medicare 3. Medicaid/State Insurance 4. Obamacare (Affordable Care Act) 5. Military (VA) 6. Private/Commercial/Employer-based/Self-insured 7. Kaiser 8. Indian Health Service 9. Healthy San Francisco 10. Other insurance | |
| 10. | Do you have a regular clinic or doctor for medical care? | 1. Yes 2. No 3. Unsure | |
| 10A | If NO to regular doctor or clinic, when was the last time you saw *any* doctor or had health care in the U.S. (besides today)? | - 1. < 6 months   2. < 6 months to a year   3. 1 year to 5 years   4. > 5 years   5. I have never seen a doctor in the U.S. | |
| 10B | If NO to regular doctor or clinic, where do you usually go when you are sick or need medical advice? | 1. An emergency department 2. A clinic 3. Urgent care center 4. Other________________________ 5. I don’t ever go anywhere 6. I have never been sick 7. Unsure | |
| 10C | If YES to regular doctor, when was the last time you saw this doctor or went to this clinic | 1. < 6 months 2. < 6 months to a year 3. 1 year to 2 years 4. > 2 years | |
| 11. | What is your primary language? | - - English   - Spanish   - Cantonese/Mandarin   - Tagalog   - Arabic   - Bengali   - Other: _________ | |
