# Supplementary material for: PROmotion of COvid-19 VA(X)ccination in the Emergency Department – PROCOVAXED: Study Protocol for a Cluster Randomized Controlled Trial
Source: Res Sq. 2022 Mar 17:rs.3.rs-1405763. Preprint. [Version 1] doi: 10.21203/rs.3.rs-1405763/v1 (PMC8936116; doi:10.21203/rs.3.rs-1405763/v1)
Supplement: Supplement 2 [file 16ece60255a8bad7e7678cec.docx]

**Subject ID _________________ Date and Time________________ CRC Initials___________ Study Arm___________**

| For NON-INTERVENTION MONTHS | | | |
| --- | --- | --- | --- |
| **#** | **Questions** | | **Answer(s)** |
| 1. | Did anyone (besides me) talk to you about Covid vaccines during your visit today?  A. Yes  B. No  C. Unsure | | 1. **Yes** 2. **No** 3. **Unsure** |
| 2. | **If YES**, who was it?   1. A doctor 2. A nurse 3. Another provider 4. A social worker 5. I don’t know | |  |
| 3. | **If YES,** did that affect how you feel about getting the Covid vaccine?   1. The message made it more likely that I will get a Covid vaccine 2. It did not affect the way I feel about getting a vaccine 3. It made it less likely that I will get a Covid vaccine | |  |
| 4. | Would you accept the Covid vaccine in the emergency department today if your doctor or provider asked you to get it? | 1. Yes 2. No 3. No but I might consider getting at some other time |  |
