# Supplementary material for: PROmotion of COvid-19 VA(X)ccination in the Emergency Department – PROCOVAXED: Study Protocol for a Cluster Randomized Controlled Trial
Source: Res Sq. 2022 Mar 17:rs.3.rs-1405763. Preprint. [Version 1] doi: 10.21203/rs.3.rs-1405763/v1 (PMC8936116; doi:10.21203/rs.3.rs-1405763/v1)
Supplement: Supplement 3 [file 43b128d2c435a596adc83ab9.docx]

**SECOND SURVEY: INTERVENTION GROUP (AFTER THE INTERVENTION)**

**Subject ID _________________ Date and Time: __________________ CRC Initials:_________**

**Study Arm________________**

| For INTERVENTION MONTHS | | |
| --- | --- | --- |
| **#** | **Questions** | **Answer(s)** |
| 1. | Did the video(s) affect the way you feel about getting a Covid vaccine? | 1. The video made it more likely that I will get a Covid vaccine 2. It did not affect the way I feel about getting a vaccine 3. It made it less likely that I will get a Covid vaccine 4. I did not watch the video |
| 2. | What would you change about the video(s)? | 1. Nothing 2. Make them shorter 3. Make them longer 4. Use a different speaker on the video. If this, who would you use?___________ 5. Other ______________________ |
| 3. | Did the information sheet affect the way you feel about getting a Covid vaccine? | 1. The sheet made it more likely that I will get a Covid vaccine 2. It did not affect the way I feel about getting a vaccine 3. It made it less likely that I will get a Covid vaccine 4. I did not look at the information sheet |
| 4. | What would you change about these printed materials? | 1. Nothing 2. Make them shorter/less information 3. Make them longer/provide more information 4. Other ______________________ |
| 5. | Did anyone (besides me) talk to you about Covid vaccines during your visit today? | a. Yes  b. No  c. Unsure |
| 6. | If YES, who was it? | 1. A doctor 2. A nurse 3. Another provider 4. A social worker 5. I don’t know |
| 7. | If YES, did that affect how you feel about getting the Covid vaccine? | 1. The message made it more likely that I will get a Covid vaccine 2. It did not affect the way I feel about getting a vaccine 3. It made it less likely that I will get a Covid vaccine |
| 8. | What would you change about these messages from the person? | 1. Nothing 2. Make them shorter/less information 3. Make them longer/provide more information 4. Other ______________________ |
| 9. | Which of the three (videos, information sheet or message from doctor) was the most useful to you? | 1. Videos 2. Print materials 3. Message from doctor 4. They were all about the same |
| 10. | Would you accept the Covid vaccine in the emergency department today if your doctor or provider asked you to get it? | 1. Yes 2. No 3. No but I might consider getting at some other time |
